# Adding tsetse control to medical activities contributes to decreasing transmission of sleeping sickness in the Mandoul focus (Chad)

**DOI:** 10.1371/journal.pntd.0005792

**Published:** 2017-07-27

**Authors:** Mahamat Hissene Mahamat, Mallaye Peka, Jean-Baptiste Rayaisse, Kat S. Rock, Mahamat Abdelrahim Toko, Justin Darnas, Guihini Mollo Brahim, Ali Bachar Alkatib, Wilfrid Yoni, Inaki Tirados, Fabrice Courtin, Samuel P. C. Brand, Cyrus Nersy, Idriss Oumar Alfaroukh, Steve J. Torr, Mike J. Lehane, Philippe Solano

**Affiliations:** 1 Institut de Recherche en Elevage pour le Développement (IRED), Ndjaména, Chad; 2 Programme National de Lutte contre la Trypanosomiase Humaine Africaine (PNLTHA), Moundou, Chad; 3 Centre International de Recherche Développement sur l’Elevage en zone Subhumide (CIRDES), Bobo-Dioulasso, Burkina Faso; 4 University of Warwick, Coventry, United Kingdom; 5 Liverpool School of Tropical Medicine, Liverpool, United Kingdom; 6 Institut de Recherche pour le Développement, UMR 177 Intertryp IRD-CIRAD, Montpellier, France; University of Florida, UNITED STATES

## Abstract

**Background:**

Gambian sleeping sickness or HAT (human African trypanosomiasis) is a neglected tropical disease caused by *Trypanosoma brucei gambiense* transmitted by riverine species of tsetse. A global programme aims to eliminate the disease as a public health problem by 2020 and stop transmission by 2030. In the South of Chad, the Mandoul area is a persistent focus of Gambian sleeping sickness where around 100 HAT cases were still diagnosed and treated annually until 2013. Pre-2014, control of HAT relied solely on case detection and treatment, which lead to a gradual decrease in the number of cases of HAT due to annual screening of the population.

**Methods:**

Because of the persistence of transmission and detection of new cases, we assessed whether the addition of vector control to case detection and treatment could further reduce transmission and consequently, reduce annual incidence of HAT in Mandoul. In particular, we investigated the impact of deploying ‘tiny targets’ which attract and kill tsetse. Before tsetse control commenced, a census of the human population was conducted and their settlements mapped. A pre-intervention survey of tsetse distribution and abundance was implemented in November 2013 and 2600 targets were deployed in the riverine habitats of tsetse in early 2014, 2015 and 2016. Impact on tsetse and on the incidence of sleeping sickness was assessed through nine tsetse monitoring surveys and four medical surveys of the human population in 2014 and 2015. Mathematical modelling was used to assess the relative impact of tsetse control on incidence compared to active and passive screening.

**Findings:**

The census indicated that a population of 38674 inhabitants lived in the vicinity of the Mandoul focus. Within this focus in November 2013, the vector is *Glossina fuscipes fuscipes* and the mean catch of tsetse from traps was 0.7 flies/trap/day (range, 0–26). The catch of tsetse from 44 sentinel biconical traps declined after target deployment with only five tsetse being caught in nine surveys giving a mean catch of 0.005 tsetse/trap/day. Modelling indicates that 70.4% (95% CI: 51–95%) of the reduction in reported cases between 2013 and 2015 can be attributed to vector control with the rest due to medical intervention. Similarly tiny targets are estimated to have reduced new infections dramatically with 62.8% (95% CI: 59–66%) of the reduction due to tsetse control, and 8.5% (95% 8–9%) to enhanced passive detection. Model predictions anticipate that elimination as a public health problem could be achieved by 2018 in this focus if vector control and screening continue at the present level and, furthermore, there may have been virtually no transmission since 2015.

**Conclusion:**

This work shows that tiny targets reduced the numbers of tsetse in this focus in Chad, which may have interrupted transmission and the combination of tsetse control to medical detection and treatment has played a major role in reducing in HAT incidence in 2014 and 2015.

## Author summary

A global programme aims to eliminate Gambian sleeping sickness (Human African Trypanosomiasis, HAT) as a public health problem by 2020. Gambian HAT is a neglected tropical disease caused by trypanosomes spread by tsetse flies and its control has relied largely on detection and treatment of human cases. In the Mandoul focus of southern Chad, regular screening of the human population (~39000 people) between 2002 and 2013 resulted in the detection and treatment of ~100 cases/year. We examined whether even better control might be achieved through the addition of vector control to medical screening. In February 2014, 2600 insecticide-treated targets (‘Tiny Targets’) were deployed in areas where tsetse were present; tsetse are attracted to the targets and on contacting it they pick up a lethal dose of insecticide. Monitoring of the tsetse population, using a network of 44 traps operated regularly between November 2013 and October 2016, showed that the mean daily catch of tsetse declined by 99.99%, from 0.7 tsetse/trap before targets were deployed to 0.005 tsetse/trap. The number of HAT cases detected by a programme of active screening also declined during this period. Mathematical modelling of the number of HAT cases reported during the period 2000–2015 suggests that 70% of the decline in cases during 2014–2015 was due to vector control. The model also suggests that the combination of these interventions may have interrupted transmission and may to lead to the elimination of sleeping sickness in the Mandoul focus by 2020. The results from Mandoul provide further empirical and theoretical evidence that the global elimination of Gambian HAT can be achieved through the integrated use of (i) case detection and treatment and (ii) vector control.

## Introduction

Human African Trypanosomiasis (HAT), also called sleeping sickness, is an endemic neglected tropical disease found in sub-Saharan Africa, caused by subspecies of *Trypanosoma brucei* transmitted by tsetse flies (*Glossina*). There is no vaccine against this lethal disease, and treatment is difficult with 1–2 weeks hospitalisation for drug treatment [1]. Most (>97%) cases of HAT are caused by *T*. *brucei gambiense*. The WHO roadmap aims to eliminate Gambian HAT as a public health problem by 2020 and reach full elimination globally by 2030 [2].

Mass screening of populations followed by diagnosis and treatment of cases has been the main method used to control Gambian HAT. These mass-screening programmes, combined with passive surveillance, have saved lives and have led to a dramatic reduction of the annual reported incidence of Gambian HAT, from > 37,000 cases/year in 1998 to <3,000 cases/year in 2015 [1, WHO Global Health Observatory data repository: http://apps.who.int/gho/data/node.main.A1636?lang=en)]. Nonetheless some foci persist despite mass screening of populations [3, 4].

The recent development and successful application of cost-effective methods—particularly so-called ‘tiny targets’ [5, 6]—offers the exciting prospect of a method that can reduce densities of tsetse in HAT foci and contribute, in association with medical interventions, to the interruption of transmission [7, 8].

An example of a persistent focus is the case of Mandoul in Southern Chad. Here, notwithstanding the progress achieved during several years of active "screen and treat" programmes, and passive surveillance, often with high coverage, the number of HAT cases reported greatly exceeded the threshold for elimination as a public health problem. In 2014, we implemented a tsetse control programme to determine whether reducing the numbers of tsetse could reduce the incidence of HAT. The entomological intervention itself was conducted using insecticide-impregnated targets that kill tsetse, whilst monitoring of vector densities was performed using traps that catch and retain the flies.

## Materials and methods

### Ethical statement

The Ministry of Health of the Republic of Chad approved the study protocols and gave administrative authorizations for the activities performed by the National Control Programme against HAT (PNLTHA: Programme National de Lutte contre la Trypanosomiase Humaine Africaine). Mass screening and treatment of HAT patients were performed according to the national Chadian HAT procedures as recommended by the World Health Organization. Information on HAT, on tsetse and tsetse control using targets and the study objectives was provided directly to individual households and more broadly via radio broadcasts (Ngor, Arab, French languages) and discussion groups organized with the Bodo district health authorities, village administrations and religious groups. Consent to participate was oral and was not mandatory. All activities were made according to the national rules of the Ministry of Health in Chad, through the national program against HAT (PM, coordinator of the NCP is one of the co-authors of the paper). These national rules do not involve written consent.

All data of the paper from participants were recorded as numbers, so anonymized, from the national program.

No human biological samples other than those required for HAT diagnosis were taken from participants in the course of this study.

### Study area

The study was performed in the Mandoul focus (ca. 8.12°N, 17.11°E) located in Southern Chad, close to the border with the Central African Republic (CAR). The study area covered part of Bodo, Beboto, Koldaga, Dilingala and Bekourou cantons and is about 840 km^2^. In this area, the mean elevation is ~400 meters and annual rainfall is between 1000 and 1200 mm/year, with two seasons: a wet season from June to October and a dry season from November to May. The landscape is characterized by woody savannah with gallery forest along the rivers. The natural vegetation is degraded in parts through agriculture activities. Within the focus lies a swamp. People pass through the swamp in the course of their daily activities. The local population comprise Ngor who are sedentary mixed crop-livestock farmers and also Arab and M’bororo (pastoralist livestock keepers). The numbers of the latter vary according to season and the availability of water and grazing in the region. Activities that bring the population into areas where tsetse are present include the cultivation of crops (sorghum, sesame, sweet potatoes), fishing, collection of wood and beekeeping. The Mandoul area is a historical focus of sleeping sickness. It was included in the “secteur de prophylaxie numéro 3” created in 1919 and was visited by the medical teams led by Gaston Muraz in 1928 [9, 10]. Two species of tsetse were historically recorded in the area: *Glossina fuscipes fuscipes* and *Glossina morsitans submorsitans* [11, 12].

### Mapping the human population

A census of the human population was conducted in 2013 to quantify the number of people and their geographical distribution. The number of persons living in households was recorded and the location recorded using a Global Positioning System (Garmin 64s). For each household, the name of the head person and the number of inhabitants were recorded. The main roads, tracks and paths leading between settlements and towards the swamp were mapped, as well as the routes used to cross the swamp.

### “T0” entomological baseline survey

Prior to the beginning of the tsetse control campaign, entomological baseline data were collected in November 2013 using 108 biconical traps deployed for 48 hours. Along the banks of the Mandoul River, traps were set in pairs, with each trap being at least 100 m apart and pairs of traps deployed at intervals of ~2 km. In addition, traps were also arranged in a transect running from the riverbank to the savannah where possible. Traps were also deployed at several jetties which were likely to be sites of human-tsetse contact.

### Target deployment

**Fig 1:**
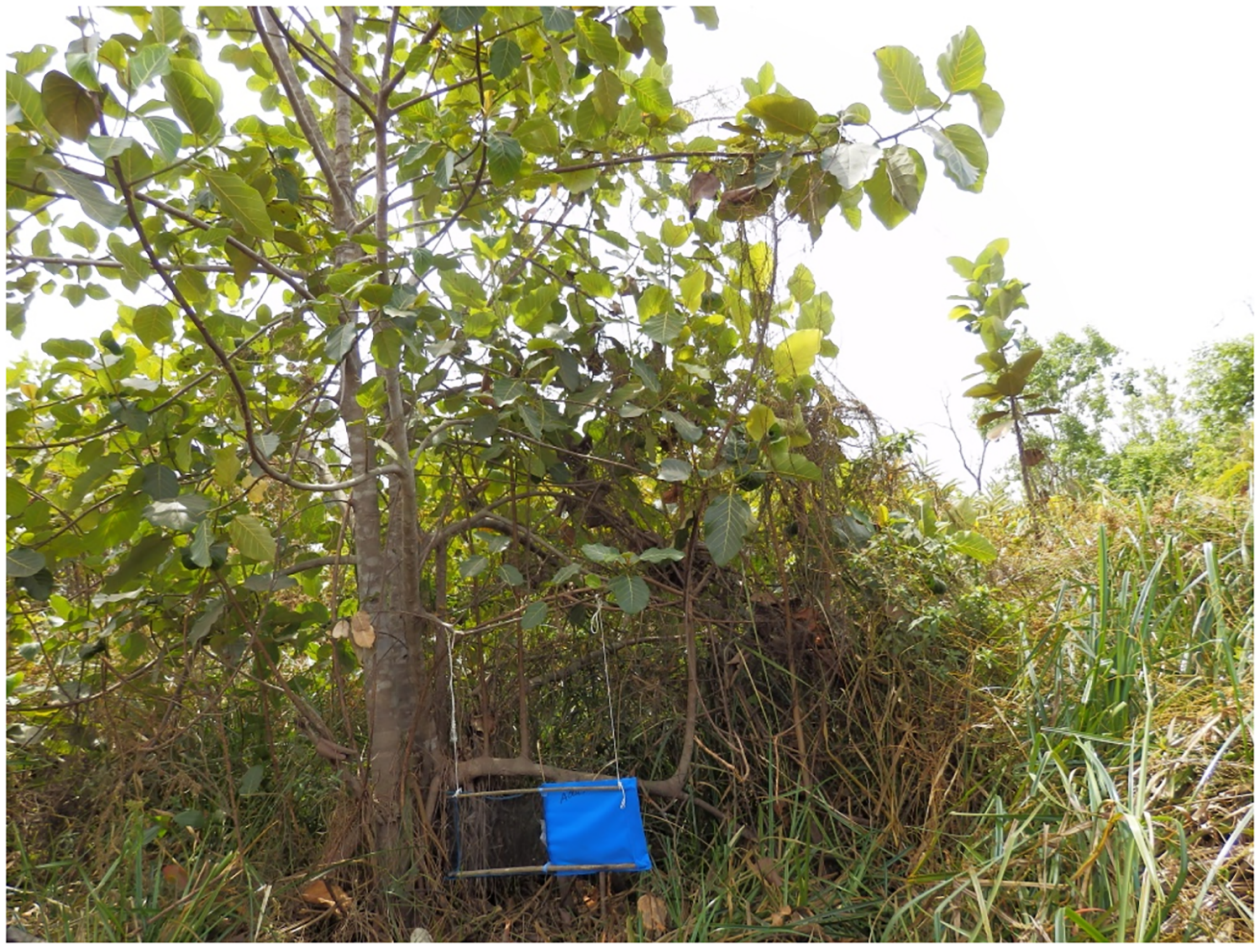
A tiny target suspended from a branch. Tsetse collide with the deltamethrin-treated target and pick up a lethal dose of insecticide [3, 8].

Tiny targets comprising 0.25m × 0.25m blue polyester flanked by 0.25m × 0.25m black polyethylene netting [6] impregnated with deltamethrin at 300mg/m^2^ were used to kill but not monitor tsetse. Targets were obtained commercially from Vestergaard-Frandsen (Lausanne, Switzerland). They were deployed in the forest gallery along the Mandoul River, in places frequented by people identified during the population census and where tsetse were caught during the pre-intervention survey. Tsetse were detected over a limited range along the Mandoul but targets were deployed up to 4 km beyond where tsetse were caught. The first deployment was done in January—February 2014, and all targets were replaced at the same period in 2015 and 2016. Targets were suspended from tree branches at 10–20 cm above the ground, using string, or erected with wooden sticks obtained locally (Fig 1).

### Entomological monitoring

Entomological monitoring was done using 44 geo-referenced sentinel traps which were selected according to both geographical coverage of the area and tsetse presence in the traps during the T0 survey. These sentinel traps were deployed for 48 h continuously at every monitoring survey to allow comparison between surveys unless stated otherwise.

Nine monitoring surveys (T1-T9) were implemented to assess changes in the catch of tsetse within the study area: the first one was undertaken in April-May 2014, 2–3 months after the deployment of targets, and thereafter during the months of March, May and October of the two following years (2015–2016). During the fourth and fifth surveys (T4, T5) respectively, additional traps and sticky traps (standard traps with the blue attractive part wrapped with transparent adhesive film; [13]) were deployed at sites where relatively high numbers of tsetse were caught during the T0 survey, in order to increase probability of catching tsetse. For the same purpose, traps were operated continuously for six consecutive days instead of the usual two during the sixth survey (T6). All surveys conducted in 2016 (T7-T9) reverted to the standard protocol with traps being operated for 48 h.

### Medical surveys

Data on HAT cases were obtained through the WHO HAT Atlas for 2000–2014 [4, 14] and also through the PNLTHA of the Republic of Chad for 2015. These epidemiological data come from both passive surveillance and active screening activities. Passive detection relies on self-presentation of symptomatic people to medical facilities. HAT cases were detected and treated according to the WHO algorithm [15], slightly modified [16] in order to allow examination of up 1500 people per day by active screening. These modifications essentially involved splitting of the medical team to examine more people. First, all screened people were tested with CATT (card agglutination test for trypanosomiasis) on whole blood, the negatives were released, and serum was taken from the positives. People positive for the CATT test done on serum up to 1/4 dilution but negative at 1/8 were released and considered as serological suspects. Parasitological examination consisted of capillary tube centrifugation (CTC), and/or direct examination of lymph nodes aspirates.

All the positive individuals to at least 1/8 dilution of the CATT test, but parasitologically negative (meaning no trypanosome detected) were considered as serological cases. Serological cases, as well as parasitological ones, where trypanosomes were observed directly through microscopic examination of blood and/or cerebrospinal fluid, were then treated with pentamidine for infections considered to be first stage, and with Nifurtimox and Eflornithine combination (NECT) for those in the second stage. Stage diagnosis was done using lumbar puncture and cerebrospinal fluid examination. In 2015 surveillance was enhanced in the Mandoul region by improving the capacity for passive screening through equipping health facilities with rapid diagnostic tests (RDTs) and upgrading confirmation and treatment centres [17].

### Statistical analysis

Comparison of catches of tsetse from traps during the different monitoring periods were analysed using a collection of generalised linear mixed-effects models (GLMEs), with Poisson error distributions and log link functions [18]. Trap sites (n = 44) were grouped into 15 different spatial locations. A baseline fixed effect was associated with each spatial location. Climatic predictors considered were precipitation and temperature and the study design was included via an indicator for tiny target (intervention) deployment and the duration of monitoring trap deployment. Trap level random effects and overdispersion were considered in the models. The best model was selected to be the one with the lowest corrected Akaike Information Criterion (AICc) and also we considered others within 2 AICc as competing hypotheses [19, 20]. All statistical analyses were carried out using the MATLAB 2015a fitglme package.

### Modelling analysis

**Fig 2:**
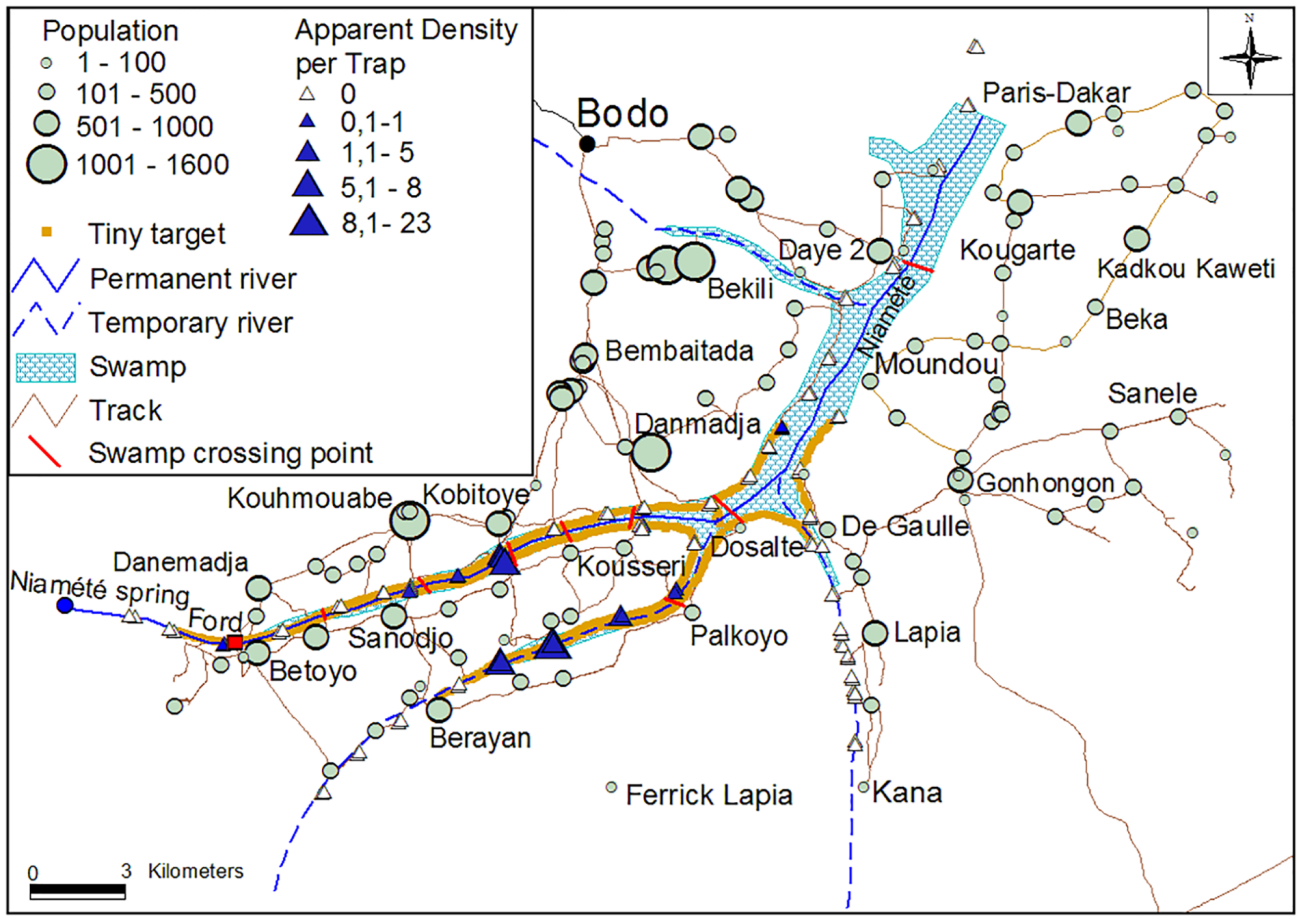
Location of the study area and distribution of human population, tsetse flies and tiny targets in the study area. This map shows the entire Mandoul focus, with the Mandoul river in blue. Target deployment started in the middle (just before the river separation in 3 branches) and the targets were mainly deployed on the two left branches.

**Fig 3:**
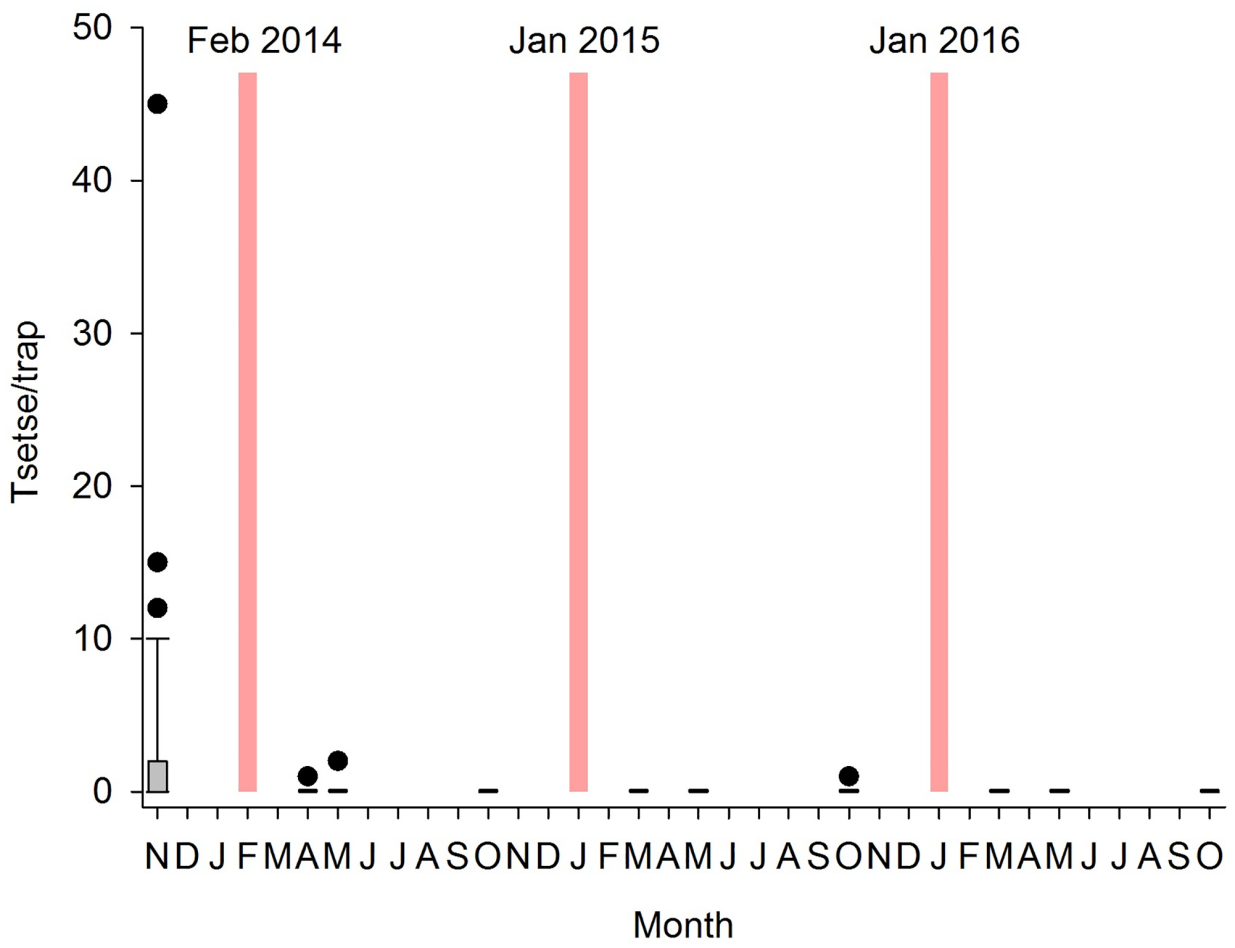
Impact of tiny targets on catch of tsetse. Black box and whiskers (with outliers shown as circles) show the numbers of tsetse caught per trap during each entomological survey (T0-T9). When no tsetse were caught during a survey, boxes appear as horizontal bars. Red bars indicate deployment of tiny targets.

In order to determine the unobservable impact of vector control upon the incidence of new infections, a previously developed mathematical model [21, 22] for Gambian HAT was modified. The mechanical model tracks human hosts in their various stages of HAT infection including stage 1 and stage 2 disease, as well as infection prevalence in the tsetse population (Fig 2 and Eq 2.1 in S1 Methodology). The model was fitted to the available epidemiological human case data using Metropolis Hastings MCMC to estimate the unknown parameters specific to this region, which include tsetse-human ratio, passive detection rate of stage 1 cases and underreporting. Model outputs of cases were stratified by their detection method (active/passive) in order to match to the data. Since infection prevalence in tsetse was unknown this could not be used for fitting, but the model can be used to provide an estimate of tsetse prevalence based on knowledge of the tsetse-host transmission cycle and fitting to the human data (Fig 3 in S1 Methodology). Case data from 2000–2013 was used to fit the model, while 2014 and 2015 were predicted based on known active screening levels continued passive surveillance and tsetse population reduction. These years were therefore used as validation years to test the predictive ability of the model.

In 2015, passive surveillance was expanded by equipping health facilities with new diagnostic tools. To account for this step-change in passive strategy in the model, it was assumed that this would speed up the detection of both stage 1 and 2 cases, and lead to less underreporting. At present, no published study has yet ascertained the quantitative impact of this type of enhanced screening upon the detection rate. The single (unstaged) data point in 2015 is unfortunately insufficient to generate robust estimates here. Therefore model predictions generate approximations for the qualitative change that would be anticipated under the model by doubling the passive detection and reporting rates for 2015 onwards.

The model provides estimates for the decline in new infections, but also the predicted impact of both medical and vector controls on future HAT reporting and transmission. Counterfactual model simulations with medical interventions but no vector control were conducted to establish the expected impact that active and passive screening would have had in the absence of vector interventions. The predictions were used to establish the feasibility of achieving elimination as a public health problem, defined as less than 1 reported case per 10,000 people, by 2020. This mathematical modelling analysis was conducted using Matlab software (see S1 Model Code). Further model details can be found in the S1 Methodology.

## Results

### Human population

A total of 114 human settlements were identified and recorded, comprising 22 encampments (<100 inhabitants), 70 hamlets (100–500 inhabitants) and 27 villages (>500 inhabitants) (Fig 2). Out of the 38 674 inhabitants counted, 1029 were located in encampments, 17 629 in hamlets, and 20 016 in villages. Therefore, in the intervention area (840 km^2^), the human population density can be estimated to be around 46 inhabitants per square kilometre. The networks of tracks between settlements and towards the swamp were recorded as well as and the main crossing tracks in the swamp (Fig 2).

### T0 survey and targets deployment

A total of 145 tsetse were caught by the 108 biconical traps during the T0 survey, all belonging to *G*. *f*. *fuscipes*, with 50 males (34.5%) and 95 females (65.5%). Apparent density in the whole area was 0.65 tsetse/trap/day, ranging from 0 to 26 tsetse/trap/day. Out of the 108 traps, 22 (20.4%) caught tsetse and they were all located on the two main branches of the Mandoul River (Fig 2, S1 Fig).

For the first deployment in 2014, 2600 targets were deployed on both sides of the river and its tributaries. These targets were replaced one year later with new ones along with an extra 108 additional targets, in order to improve the area coverage (Fig 2), giving a total number of 2708 targets. Out of the 840 km^2^ of the intervention area of the Mandoul focus, the actual area on which targets were deployed represents 45 km^2^, corresponding to the gallery surrounding the portion of the Mandoul River where tsetse were found. Hence the overall target density can be calculated either as 3.2 targets/km^2^ if based on the whole intervention area, or 60 targets per linear km if only considering the riverine system.

### Impact of tiny targets on tsetse densities

The total catch of tsetse from 44 sentinel traps was 145 tsetse (1.62 flies/trap/day). At the first evaluation post target deployment (T1, 2 months later), only 2 tsetse were caught (0.02 tsetse/trap/day) were observed (Fig 3). The same density was again observed during the second evaluation (T2) but thereafter no tsetse was caught during the next three successive surveys (T3-T6), even with the additional traps and sticky traps deployed to increase the probability of capture. During the sixth survey (T6, October 2015), trapping duration was extended to six consecutive days and one tsetse was caught. No tsetse were caught during the three surveys conducted in 2016 (T7-T9).

Statistical analysis using GMLE resulted in two selected models differing by only one effect (see Section 1 in S1 Methodology for more detailed results). The best model had only location specific baselines, the pre-target deployment indictor (*p*<10^−18^, coeff = 5.3 i.e. a predicted 99.5% reduction due to tiny target intervention) and an overdispersion effect. The other selected model also included a precipitation fixed effect, but the pre-target deployment indicator remained strongly significant (*p*<10^−11^, coeff = 5.4).

### Modelling the impact of tiny targets on new HAT infections

**Fig 4:**
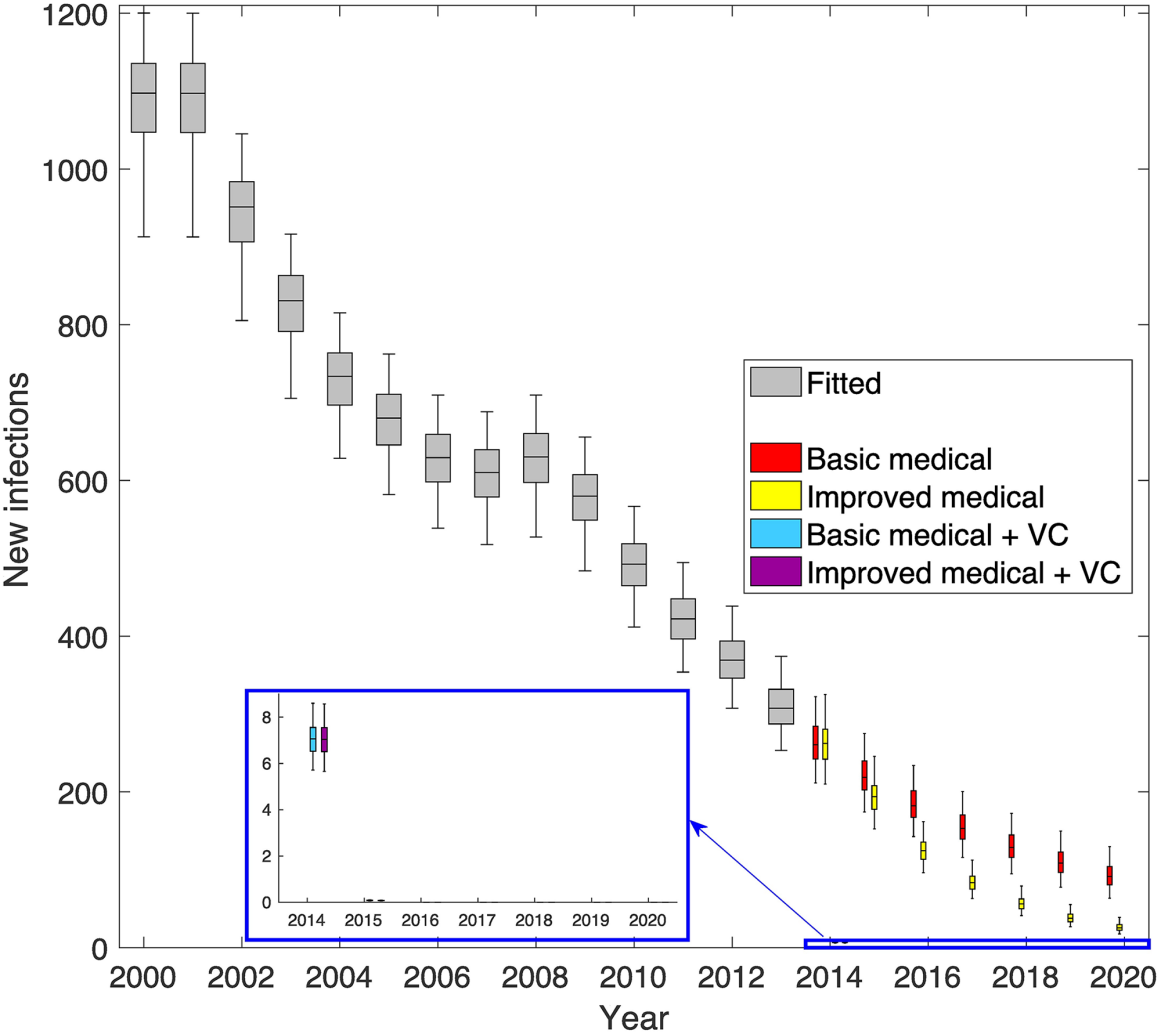
Inferred level of underlying transmission from modelling. The grey box plots show the estimated numbers of new infections in humans for each year (2000–2013). Coloured boxes denote the predicted number of new infections from 2014 onwards under four different strategies including combinations of either basic or enhanced medical strategies and vector control. Improved medical strategies had increases to the passive detection and reporting rates from 2015, whilst vector control strategies began in 2014. Purple boxes denote the strategy that took place with both improvements to passive screening and tsetse control.

**Fig 5:**
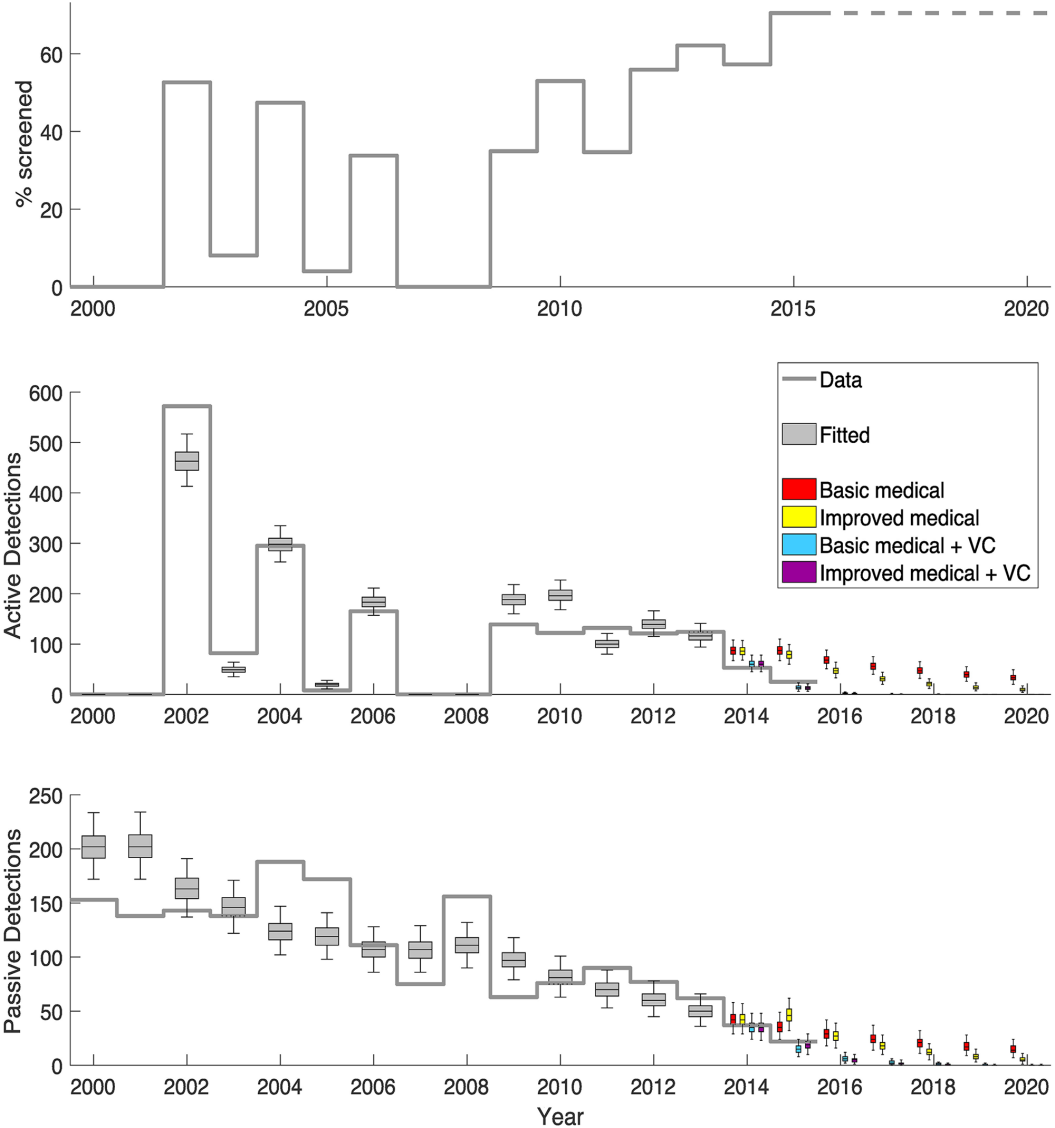
Model fit to data. The top graph shows the annual level of active screening achieved in the focus. The middle and bottom graphs show the observed active and passive case detections as a solid grey line with the model fit (years 2000–2013) displayed as grey box and whisker plots. The model projections of the four strategies for 2014 onwards are shown as coloured box plots and include combinations of either basic or enhanced medical strategies and vector control. Improved medical strategies had increases to the passive detection and reporting rates from 2015, whilst vector control strategies began in 2014. Purple boxes denote the strategy that took place with both improvements to passive screening and tsetse control.

Model fitting to human case data indicates that active screening has played a major role in the reduction of new HAT infections between 2000 and 2013. Over this time there is an estimated 71.5% (95% CI: 67–75%) reduction in new transmissions (see Fig 4). This trend is not so apparent in the detected HAT cases due to high fluctuation in screening levels during that time period, leading to a variable detection rate. Despite this ambiguity, it is noted that from 2009 onwards, detected cases remained at relatively low levels despite annual screening at moderate levels (see Fig 5). It is seen that passive screening plays a variable role in case detection, and the model can capture this trend. For example with more cases being detected by passive surveillance in 2008 following no active screening in 2007/2008.

To assess the robustness of model predictions, an additional fit was performed using only epidemiological data for 2000–2006, and used 2007–2013 as validation years without vector control and 2014–2014 as validation years with vector control. The model dynamics obtained were very similar to those presented here, despite using only the first half of the data set (see Section 3.2 in S1 Methodology for detailed analysis).

Projecting 2014 and 2015 was performed using four different strategies: (1) basic medical, using known levels of active screening and assuming passive detection continued unchanged in 2015, (2) improved medical, using known active screening and assuming passive detection and reporting rates doubled in 2015, (3) basic medical and vector control, the same as basic medical with vector control from 2014, and (4) improved medical and vector control, the same as improved medical with vector control from 2014. Strategy 4 represents the strategy that occurred in 2014–2015 and is coloured purple in Figs 4 and 5. It is seen that, using the actual strategy, the model captures the trend in reported cases for 2014/2015. Comparing these years to the counterfactual strategies (1–3) predictions demonstrates the additional benefit of having added tsetse control and/or enhanced passive surveillance to the existing medical intervention.

Mathematical modelling estimates that, without vector control, there would have been 129 (95% CI, 105–157) and 123 (95% CI, 97–150) cases per year in 2014 and 2015 respectively with the reduction due to active screening. In reality only 90 and 47 cases were observed (in 2014 and 2015) when a combined vector and enhanced medical strategy took place; in 2013 there were 186 cases. Modelling results indicate that 29.6% (95% CI: 5–49%) of this two-year decline was through active screening, and the remaining 70.4% (95% CI: 51–95%) was due to vector control. In 2015, the new passive strategy was predicted to result in slightly more case reporting than the basic medical strategy and so was not attributed to this particular decline.

Likewise, new infections are calculated to have fallen dramatically. The model surmises that transmission fell from around 311 new infections per year in 2013 to a mean of 7 and 0 new infections per year in 2014 and 2015 respectively. Over these two years, 28.7% (95% CI: 25–33%) of this decline is attributed to active screening, 8.5% (95% CI: 8–9%) to passive and 62.8% (95% CI: 59–66%) to vector control.

Projecting forwards to 2020 using the model gives an optimistic forecast if vector control and screening continue at the 2015 level. Continuing this combined strategy is predicted to result in fewer than 2 annually reported cases and zero transmission in 2020; this strategy appears sufficient to reach elimination as a public health problem by 2018 and full elimination over a decade in advance of the WHO 2030 goal. It is noted that the model predicts this will occur with or without enhanced passive screening. Under the counterfactual strategy without vector control, the reported cases and transmission may have continued to decline steadily, reaching around 47 (95% CI: 31–67) reported cases in 2020 without enhanced passive screening; although this would have still been substantially higher than the 3.9 reported cases threshold that is necessary to achieve elimination as a public health problem in the focus. Results from the counterfactual strategy without vector control but with enhanced passive detection, predicted that there would have been even fewer reported cases compared to the basic medical strategy, with 20 (95% CI: 7–25) reported cases in 2020, however this would still be above the target threshold.

## Discussion

This study shows that adding tsetse control to the ‘screen and treat’ strategy had a marked impact on the transmission of sleeping sickness in the Mandoul focus. This echoes the recent findings from Guinea where tsetse control also resulted in a significant decrease of the incidence of HAT [3].

The total number of cases diagnosed and treated (1143 cases since 2009 out of a population of 38 674 people) suggests that up to 3.0% of the people in this area have had sleeping sickness. A huge medical effort was conducted in this focus with 142 467 people being screened between 2009 and 2015. Given the population number is ~38 674, it means that on average, every individual in the area was screened nearly four times during this period. Despite this effort, a substantial decrease in HAT reporting occurred only when vector control was added to the medical strategy. In 2014, there was a decrease in the stage 1 to stage 2 detection ratio for both active and passive surveillance. For active screening this ratio was 1.5 stage 1 cases for every stage 2 case which decreased from 2.7 in 2012 and 2013 when a similar level of active screening was achieved. For passive surveillance there were 0.16 stage 1 cases per stage 2 case in 2014, which decreased from 0.22 and 0.24 in 2012 and 2013 respectively. This relative decline in the stage 1 to stage 2 case ratio supports the hypothesis that vector control has reduced new transmissions, and is expected to continue to decline over the coming years whilst tsetse control remains in place, although the improved passive surveillance that began in 2015 could skew this ratio. Modelling supports the assertion that tsetse control has greatly impacted past reporting and transmission, and will continue to do so whilst the fly population remains supressed.

It is acknowledged that there are several assumptions that effect the results generated by the mathematical modelling results presented here, many of which have been discussed elsewhere [21, 22]. Given the relatively small size of the Mandoul focus, it is unlikely that spatial effects would alter the presented results, however temporal heterogeneities arising from unknown dates of screenings could explain some of the discrepancies between reported HAT cases and model outputs. Furthermore, the dynamic model does not take importations from other areas into account, which could result in other small differences between the model and data. By additional analysis which used 2007–2015 as validation years (see S1 Methodology), the model seems to perform well at mid-range prediction, providing evidence for the model’s forecasting ability and justification for its use here as a tool to evaluate intervention strategies.

One particular challenge was to estimate the impact of new enhanced medical strategies, which began in the region in 2015, on case detection and reporting. Unfortunately there was little information with which to accurately parameterise the mathematical model to account for this change in strategy, although the presented results demonstrate the expected qualitative impact that this has had, and should continue to have, in Mandoul. The modelling results demonstrate that it very possible that improved screening actually increased the number of passively detected cases in 2015, but would be expected to lead to reduced cases in subsequent years by reducing transmission. As there was uncertainty surrounding this strategy, results without improved screening were also shown for comparison. Future work using other data sets could help to better inform model parameterisation of this type of enhanced medical strategy.

For vector control, the lack of a control (non-intervention) area is a limitation for the study. However, the aim of the intervention was to protect the people of Mandoul from sleeping sickness. This is an isolated focus without a comparable area, which could have served as an appropriate control. In the absence of a control, empirical data, gathered before and after entomological intervention, has been used in conjunction with models to estimate its relative impact.

The vector in the Mandoul area is *G*. *f*. *fuscipes* as reported previously [23]. *G*. *morsitans submorsitans* was captured by surveys conducted 20 years ago [11], but none was caught during the current surveys, or previous ones in the area [23]. This confirms its disappearance in places where human encroachment is high and where wildlife disappears, as reported elsewhere [24, 25].

In the Mandoul focus, the medical ‘screen and treat’ strategy saved many lives but the addition of vector control has driven the incidence to even lower levels (0.38%) and this may have interrupted transmission of *T*. *brucei gambiense*. Why was this incidence not achieved without vector control? Among several possible causes, the geographical situation and human behaviour in this area may be a key parameter, especially human mobility. The human population frequently crosses the tsetse-infested swamp. The population between the two banks come from the same ethnic group and often belong to the same families. It means that for any social event, people need to cross the swamp. Moreover, there is an important local market and facilities (healthcare, veterinary, school, local government) in the town of Bodo which is visited frequently by local people, many of whom must cross the swamp to reach Bodo.

The rapid and drastic decrease in catch of tsetse suggests that tiny targets can control *G*. *f*. *fuscipes* in this setting. The small size of the area, well defined with limited habitat for tsetse and its relative isolation with low reinvasion pressure may have contributed to the success of the intervention. This contrasts with two other foci where tiny targets have been recently used to control tsetse. In the Boffa focus of Guinea, extensive and difficult-to-access mangrove made vector control (*G*. *palpalis* there) much more difficult [5, 26]. Similarly in the Arua focus of northern Uganda, the widely distributed population of *G*. *f*. *fuscipes* was not driven to the low (i.e., close to zero) levels seen in Chad [8].

Present results further demonstrate the efficacy of combining medical interventions with vector control to halt *T*. *b*. *gambiense* transmission, in certain epidemiological settings. Unlike medical interventions, which reduce disease duration and consequently decrease the number of new infections, tsetse control directly prevents people from becoming infected. As neither vaccine nor chemoprophylaxis for HAT exist this provides a valuable protective tool. As in Chad, Uganda and Guinea, this strategy can be applied in other foci where HAT still exists despite medical surveys and treatment of cases. While the use of tiny targets was effective in this operation, other methods may be appropriate elsewhere. For instance, many cattle are present in Mandoul. These animals, owned by sedentary farmers and more mobile pastoralists, are at risk of animal African trypanosomiasis [23] and hence controlling tsetse, using targets and/or treating cattle with insecticide, will also have a positive consequence on both animal and human health. This means that with minimal additional efforts, expanding this one health approach could benefit to people and their animals in this area, as certainly in other areas of the same type [27].

## Supporting information

S1 Methodology Additional information on statistical and mathematical modelling methodology. (PDF)

S1 Model Code MATLAB code used to generate mathematical model outputs. (M)

S1 Fig Portion of the Mandoul River where tsetse flies were caught. (TIF)
