# Supplementary material for: Adding tsetse control to medical activities contributes to decreasing transmission of sleeping sickness in the Mandoul focus (Chad)
Source: PLoS Negl Trop Dis. 2017 Jul 27;11(7):e0005792. doi: 10.1371/journal.pntd.0005792 (PMC5549763; doi:10.1371/journal.pntd.0005792)
Supplement: S1 Methodology — (PDF) [file pntd.0005792.s001.pdf]

Supplementary Information
Adding tsetse control to medical activities contributes to decreasing transmission of sleeping sickness in the Mandoul focus (Chad)

Mahamat Hissene Mahamat[1], Mallaye Peka[2], Jean-Baptiste Rayaisse*[3], Kat S. Rock[4], Mahamat Abdelrahim Toko[1], Justin Darnas[2], Guihini Mollo Brahim[1], Ali Bachar Alkatib[†1], Wilfrid Yoni[3], Inaki Tirados[5], Fabrice Courtin[6], Samuel P. C. Brand[4], Cyrus Nersy[1], Idriss Oumar Alfaroukh[1], Steve J. Torr[4,5], Mike J. Lehane[5], Philippe Solano[6]

* Corresponding author: jbrayaisse@hotmail.com
† In memory of Ali Bachar Alkatib, killed by bees during the field work reported here.
1 Institut de Recherche en Elevage pour le Développement (IRED), Ndjaména, Chad
2 Programme National de Lutte contre la Trypanosomiase Humaine (PNLTHA), Ndjaména, Chad
3 Centre International de Recherche Développement sur l'Elevage en zone Subhumide (CIRDES), Bobo-Dioulasso, Burkina Faso
4 University of Warwick, Coventry, United Kingdom
5 Liverpool School of Tropical Medicine, Liverpool, United Kingdom
6 Institut de Recherche pour le Développement, UMR 177 Intertryp IRD-CIRAD, 34398 Montpellier, France

S1 Statistical model selection

To assess the impact of the tiny target intervention on the tsetse population size a statistical analysis was performed using an ensemble of generalised linear mixed effects models (GLMMs) with the number of tsetse flies capture per trapping event as the response variable. As is standard with count data Poisson error distributions were assumed and a log link function was used to transform the linear predictor into a Poisson mean prediction. A plausible range of fixed and random effect were considered which included both intervention impact, climatic predictors and spatial location. A full list of the considered effects is given in Table S1 and a list of considered GLME models and their corresponding corrected Akaike Information Criterions (AICcs) is presented in Table S2.

The model with the lowest AICc was found to be Model G, which included the location specific baselines, the target deployment indictor as fixed effects and overdispersion. The other selected model, Model F, was chosen as it was within 2 AICc of the best model; it was identical to model G except that mean daily precipitation over the 2 weeks before trap collection was included as a fixed effect.. Both selected models (G and F) found that the target deployment indicator was highly significant ($P < 10^{-18}$ and $P < 10^{-11}$ respectively). These results are evidence that the intervention was impactful as the selected models were strongly selected over the null statistical model (Model I). Coefficients and standard errors of models F and G are given in Tables S3 and S4 respectively.

Table S1: Possible fixed and random effects list

Name	Description
$\mathbb{1}_{T0}$	Indicator for whether the catch occurred in T0 (before target deployment) or not (after target deployment)
Temp	Temperature (°C) given as the mean over the monitoring period and the proceeding week as measured from the closest weather station (Moundou) [S11]
Rain	Rainfall (mm) given as the mean over the monitoring period and the proceeding week as measured from the closest weather station (Moundou) [S11]
Capture	Specific capture (i.e. specific trap at given sampling period, 396 captures)
Trap	Specific trap location (44 traps)
Loc	Grouped spatial locations of traps (15 locations)
$\mathbb{1}_{6days}$	Indicator for whether the trap was deployed for 6 days or not (2 days)

Table S2: GLME model selection

	Model	AICc
A	Number $\sim$ 1 + $\mathbb{1}_{T0}$ + Rain*Temp+ loc + $\mathbb{1}_{6days}$ + (1|Capture) + (1|Trap)	203.3
B	Number $\sim$ 1 + $\mathbb{1}_{T0}$ + Rain*Temp+ loc + $\mathbb{1}_{6days}$ + (1|Capture)	201.6
C	Number $\sim$ 1 + $\mathbb{1}_{T0}$ + Rain*Temp - Temp + loc + $\mathbb{1}_{6days}$ + (1|Capture)	200.1
D	Number $\sim$ 1 + $\mathbb{1}_{T0}$ + Rain + Temp + loc + $\mathbb{1}_{6days}$ + (1|Capture)	200.1
E	Number $\sim$ 1 + $\mathbb{1}_{T0}$ + Rain + loc + $\mathbb{1}_{6days}$ + (1|Capture)	198.4
F	Number $\sim$ 1 + $\mathbb{1}_{T0}$ + Rain + loc + (1|Capture)	196.8
G	Number $\sim$ 1 + $\mathbb{1}_{T0}$ + loc + (1|Capture)	195.0
H	Number $\sim$ 1 + $\mathbb{1}_{T0}$ + (1|Capture)	224.2
I	Number $\sim$ 1 + (1|Capture)	249.87

Table S3: Model F fixed effects coefficients

Name	Estimate	Standard error	p Value
Intercept	-5.65	1.48	0.000151
Loc: Taboumti	-15.7	2020	0.994
Loc: Bekono	-15.7	2020	0.994
Loc: Dedaye	-15.7	2020	0.994
Loc: Danmandja	1.64	1.46	0.260
Loc: Sananga	-15.7	2020	0.994
Loc: Daraibe	2.30	1.40	0.102
Loc: Dankou	2.73	1.39	0.0500
Loc: Betoyo 2	1.27	1.37	0.357
Loc: Deb Sanadjio 2	-0.737	1.77	0.678
Loc: Kobitoye	0.716	1.48	0.630
Loc: Danmandja1	-15.7	2020	0.994
Loc: Kousseri	-0.737	1.77	0.678
Loc: Lapia	-15.7	884	0.986
$\mathbb{1}_{T0}$	5.40	0.734	1.20×10^{-12}
Rain	0.0475	0.299	0.874

Table S4: Model G fixed effects coefficients

Name	Estimate	Standard error	p Value
Intercept	-5.57	1.40	7.73×10^{-5}
Loc: Taboumti	-15.6	1870	0.993
Loc: Bekono	-15.6	1870	0.993
Loc: Dedaye	-15.6	1870	0.993
Loc: Danmandja	1.64	1.46	0.260
Loc: Sananga	-15.6	1880	0.993
Loc: Daraibe	2.29	1.40	0.102
Loc: Dankou	2.73	1.39	0.0501
Loc: Betoyo 2	1.27	1.37	0.357
Loc: Deb Sanadjio 2	-0.737	1.77	0.678
Loc: Kobitoye	0.716	1.48	0.630
Loc: Danmandja1	-15.6	1880	0.993
Loc: Kousseri	-0.737	1.77	0.678
Loc: Lapia	-16.0	1060	0.988
$\mathbb{1}_{T0}$	5.33	0.555	1.08×10^{-19}

An observed power calculation was performed retrospectively [S16]. Because closed form distribution functions are unavailable for GLMMs the methodology of Johnson *et al.* [S9] was followed in sampling from the GLMM models that best fitted the data in order to construct an estimate of the power of the study as a function of the critical significance level (α). This was done by:

1. Resampling a synthetic capture data set 10,000 times from each of the best two GLMMs.

2. Refitting each of the best two GLMMs for each synthetic data set and calculating the *p*-value associated with the null hypothesis that the intervention effect was 0.

3. The observed power estimate for any given critical significance level, α, is the relative frequency of *p*-values below the significance level. Confidence intervals for the observed power were constructed using bootstrapping.

Using "highly significant" ($\alpha = 0.001$) as the critical value for rejecting the null hypothesis (that the intervention did nothing) in favour of the intervention having an effect, implied that an observed power for Model F is 0.9739 [0.9706, 0.9768] and for Model G observed power is 0.9946 [0.9930, 0.9960]. Figure S1 shows a plot of the observed power of the study against α (the critical value chosen for rejecting the null hypothesis). The exceptionally high retrospective observed power for the study is unsurprising because the intervention effect estimated from the study was large and highly statistically significant.

A couple of statistical points should be noted: (i) that the hypothesis test for rejecting the null hypothesis is two-sided due to the MATLAB method of calculating the *p*-value, however each re-fitted GLMM found the intervention effect as negative for the expected number of tsetse flies trapped and (ii) the effect of clustering in the underlying data on statistical prediction is accounted for within the GLMM framework by allowing cluster specific fixed and/or random effects, e.g. the trap location specific fixed effects in the best fitting models in this study (see Bolker et al [S1] for a discussion of GLMM best-practice in ecological modelling). Therefore, effective sample size corrections (e.g. the design effect) and measures of clustered data relatedness (e.g. intraclass correlation) are unnecessary in this context.

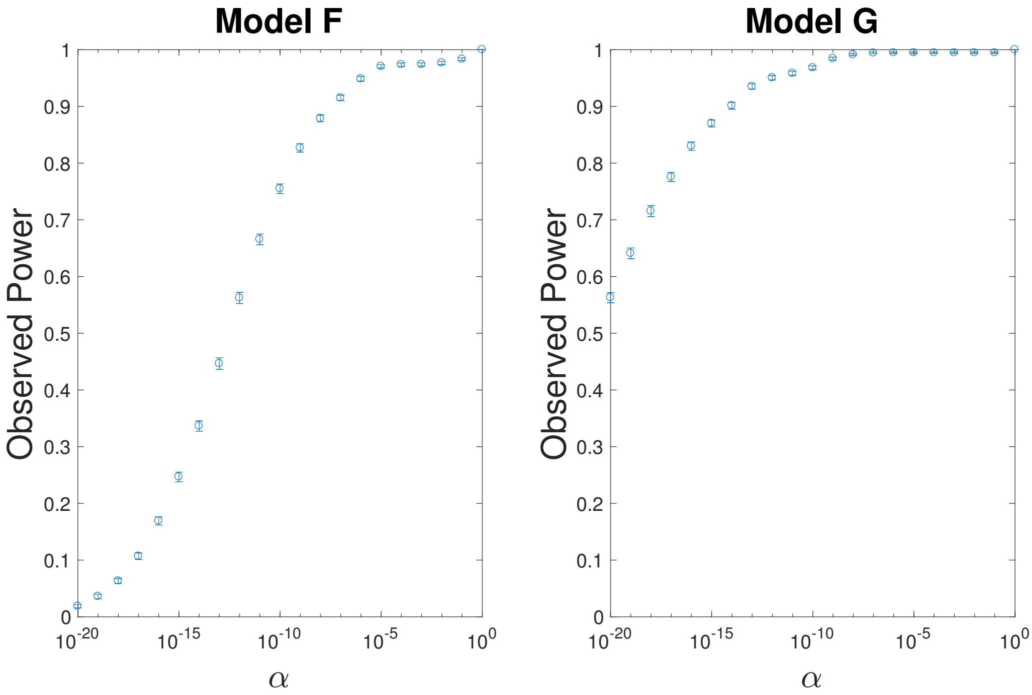

Figure S1: Observed power by significance level (α) under the two best GLMMs, Model F and Model G.

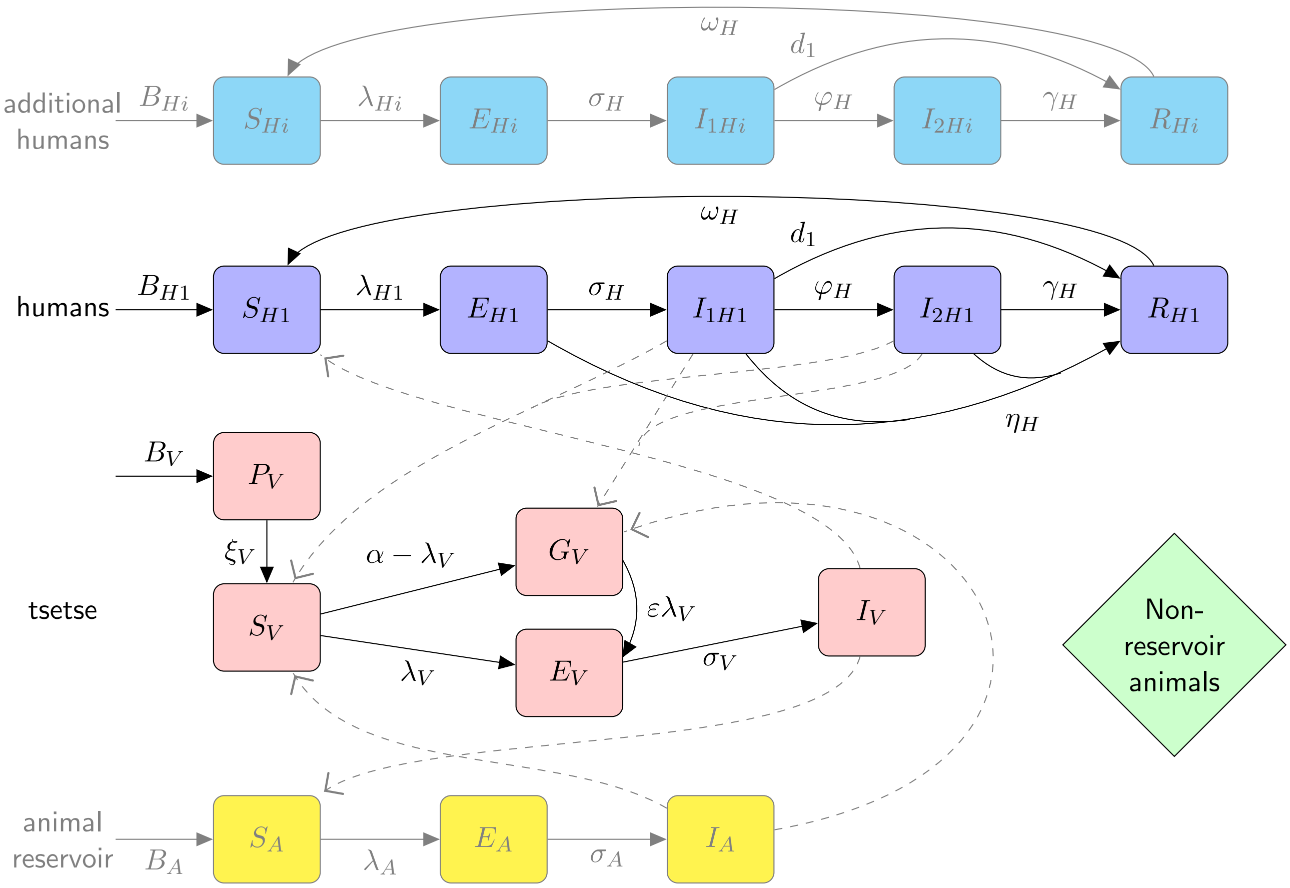

Figure S2: Multi-host model of HAT with one host species able to confer HAT (humans), a further non-reservoir species (others) and tsetse. Human hosts follow the progression which includes an infectious stage 1 disease, I_{H1}, infectious stage 2 disease, I_{H2}, and a non-infectious (due to hospitalisation) disease, R_1. P_V are pupal stage tsetse which emerge into unfed adults. Unfed tsetse are susceptible, S_V, and following a blood-meal become either exposed, E_V, or have reduce susceptibility to the trypanosomes, G_V. Tsetse select their blood-meal from one of the two host species dependant upon innate feeding preference, s, and relative host abundance, k. Any blood-meals taken upon "other" hosts do not result in infection. The transmission of infection between humans/tsetse and reservoirs/tsetse is shown by grey paths. This figure is adapted from the original model schematic [S13].

S2 Dynamic model structure

S2.1 Model description and equations

The HAT model equations are given below (S2.1) and correspond with the compartmental diagram, Fig S2, and the model outputs, Fig S3. The model is largely the same as that presented by Rock *et al* [S13] and [S14].

Human hosts are assumed to be in one of four distinct classes: either low-risk and randomly participate in screening (subscript $H1$), high-risk and random participation ($H2$), low-risk and never participate in screening ($H3$) or high-risk and never participate. Tsetse bites are assumed to be taken on humans or animals. The model incorporates reservoir animals which can become infected and assumes that the remainder of the bites are taken on non-reservoir animal species which do not need to be explicitly modelled.

The free parameters, $k_1, k_2, k_3, k_4, m_{eff}, r, f_A, N_A, u, d_1$ (denoted by "Fitted' in the table) were fitted to data using MCMC.

$$\text{Humans}\begin{cases}\frac{\mathrm{d}S_{Hi}}{\mathrm{d}t} &= \mu_H N_{Hi} + \omega_H R_{Hi} - \alpha m_{\text{eff}} f_i \frac{S_{Hi}}{N_{Hi}} I_V - \mu_H S_{Hi}\\ \frac{\mathrm{d}E_{Hi}}{\mathrm{d}t} &= \alpha m_{\text{eff}} f_i \frac{S_{Hi}}{N_{Hi}} I_V - (\sigma_H + \mu_H) E_{Hi}\\ \frac{\mathrm{d}I_{1Hi}}{\mathrm{d}t} &= \sigma_H E_{Hi} - (\varphi_H + d_1 + \mu_H) I_{1Hi}\\ \frac{\mathrm{d}I_{2Hi}}{\mathrm{d}t} &= \varphi_H I_{1Hi} - (\gamma_H + \mu_H) I_{2Hi}\\ \frac{\mathrm{d}R_{Hi}}{\mathrm{d}t} &= d_1 I_{1Hi} + \gamma_H I_{2Hi} - (\omega_H + \mu_H) R_{Hi}\end{cases}$$

$$\text{Animals}\begin{cases}\frac{\mathrm{d}S_{A}}{\mathrm{d}t} &= \mu_A N_A - \alpha m_{\text{eff}} f_A \frac{S_A}{N_A} I_V - \mu_A S_A\\ \frac{\mathrm{d}E_{A}}{\mathrm{d}t} &= \alpha m_{\text{eff}} f_A \frac{S_A}{N_A} I_V - (\sigma_A + \mu_A) E_A\\ \frac{\mathrm{d}I_{A}}{\mathrm{d}t} &= \sigma_A E_A - \mu_A I_A\end{cases}$$

$$\text{Tsetse}\begin{cases}\frac{\mathrm{d}P_{V}}{\mathrm{d}t} &= B_V N_V - (\xi_V + \frac{P_V}{K}) P_V\\ \frac{\mathrm{d}S_{V}}{\mathrm{d}t} &= \xi_V \mathbb{P}(\text{survive pupal stage}) P_V - \alpha p_V \big(\sum_i f_i \frac{(I_{1Hi} + I_{2Hi})}{N_{Hi}} + f_A \frac{I_A}{N_A} \big) S_V\\ & \quad -\mu_V S_V\\ \frac{\mathrm{d}E_{1V}}{\mathrm{d}t} &= \alpha(1 - f_T(t)) p_V \big(\sum_i f_i \frac{(I_{1Hi} + I_{2Hi})}{N_{Hi}} + f_A \frac{I_A}{N_A} \big) (S_V + \varepsilon G_V)\\ & \quad -(3\sigma_V + \mu_V + \alpha f_T(t)) E_{1V}\\ \frac{\mathrm{d}E_{2V}}{\mathrm{d}t} &= 3\sigma_V E_{1V} - (3\sigma_V + \mu_V + \alpha f_T(t)) E_{2V}\\ \frac{\mathrm{d}E_{3V}}{\mathrm{d}t} &= 3\sigma_V E_{2V} - (3\sigma_V + \mu_V + \alpha f_T(t)) E_{3V}\\ \frac{\mathrm{d}I_{V}}{\mathrm{d}t} &= 3\sigma_V E_{3V} - (\mu_V + \alpha f_T(t)) I_V\\ \frac{\mathrm{d}G_{V}}{\mathrm{d}t} &= \alpha(1 - f_T(t)) \Big(- p_V \big(\sum_i f_i \frac{(I_{1Hi} + I_{2Hi})}{N_{Hi}} + f_A \frac{I_A}{N_A} \big) \Big) S_V\\ & \quad -\alpha \Big(f_T(t) + (1 - f_T(t)) p_V \varepsilon \big(\sum_i f_i \frac{(I_{1Hi} + I_{2Hi})}{N_{Hi}} + f_A \frac{I_A}{N_A} \big) G_V \big)\\ & \quad -\mu_V G_V\end{cases} \tag{S2.1}$$

The function which describes the probability of both hitting a target and dying is time dependent (days) from when the targets where placed:

$$f_T(t) = f_{\text{max}} \left(1 - \frac{1}{1 + \exp(-0.068(\text{mod}(t, 365) - 127.75))} \right) \tag{S2.2}$$

and f_{max} is chosen to be 0.42 so that the tsetse population is at the observed 99% reduction four months after deployment.

$N_H = \sum_i N_{Hi}$ and the actual number of vectors is $S_V, E_{1V}, E_{2V}, E_{3V}$ and I_V multiplied by N_V/N_H.

$\sum_i f_i = f_H$ i.e. the total proportion of tsetse bites taken on humans. s_i is the relative availability/attractiveness of different host types, so for the 4 different humans types (low/random participant, high/random, low/non-participant, high/non), where high risk humans are r-fold more likely to receive bites, $s = (1, r, 1, r)$. The f_i's are calculated using $f_i = \frac{s_i N_{Hi}}{\sum_j s_j N_{Hj}}$.

S2.2 Parameter table

S2.3 Output

The compartmental ODE model is simulated to compute the disease dynamics in humans, animals and tsetse (see Fig S3). The total annual passive reported cases for year, T is calculated by integrating over the new hospitalisations from self-presentation multiplied by the reporting parameter, u, to compensate for underreporting of passive cases:

$$P_M = u \sum_i \int_T^{T+1} d_1 I_{1Hi}(t) + \gamma_H I_{2Hi}(t) \, \mathrm{d}t \tag{S2.3}$$

where $i \in$ all human types), whereas the active number of reported cases is given as:

$$\begin{aligned} A_M = & \textstyle\sum_j \text{ proportion screened} \\ & \quad \times \text{ test sensitivity} \\ & \quad\quad \times \text{ compliance} \\ & \quad\quad\quad \times (I_{1Hj}(T) + I_{2Hj}(T)) \\ + & \quad \text{proportion screened} \\ & \quad\quad \times (1 - \text{test specificity}) \\ & \quad\quad\quad \times \text{ compliance} \\ & \quad\quad\quad\quad \times (N_{Hj}(T) - I_{1Hj}(T) - I_{2Hj}(T)) \end{aligned} \tag{S2.4}$$

where $j \in$ random participants. The number of reported cases seen under the model is also shown in Fig S3.

S3 Model analysis

S3.1 Fitting and selection

Data was provided at an annual temporal resolution, however it is known that many years had multiple rounds of active screening. Since the number and timing of screenings is largely unclear, an algorithm which estimates these was used in this analysis and is summarised in Table S6.

The model was fitted to human case data between 2000 and 2013. Out of these years, 2000–2004 and 2006 had no (or very little) staging information for passive cases. For active detections or passive detections in other years, any cases without staging information were assumed to occur with the same proportion of stage 1 and 2 to those with staging information. Using this the model could be fitted to staged case data using the likelihood function:

$$\begin{aligned} LL(\theta|x) & \propto \log(\mathbb{P}(x|\theta)) \\ & = \textstyle\sum_{i=2000}^{2013} \ln \left[Bin(A_{1D}(i); z(i)N_H, \frac{A_{1M}(i)}{N_H}) \right] + \ln \left[Bin(A_{2D}(i); z(i)N_H, \frac{A_{2M}(i)}{N_H}) \right] \\ & \quad + \textstyle\sum_{i=j} \ln \left[Bin(P_D(i); N_H, \frac{P_M(i)}{N_H}) \right] \\ & \quad\quad + \textstyle\sum_{i=k} \ln \left[Bin(P_{1D}(i); N_H, \frac{P_{1M}(i)}{N_H}) \right] + \ln \left[Bin(P_{2D}(i); N_H, \frac{P_{2M}(i)}{N_H}) \right] \end{aligned} \tag{S3.1}$$

where $j = \{2000, 2001, 2002, 2003, 2004, 2006\}$, $k = \{2000, ..., 2013\} \setminus j$. The model takes the parameterisation $\theta = (R_0, r, k_1, k_2, k_3, k_4, k_A, f_A, d_1, u)$, x is the data, $Bin(m; n, p)$ is the binomial probability of obtaining m successes out of n trials with probability p. $A_{1D}(i), A_{2D}(i), P_{1D}(i), P_{2D}(i)$ are the number of stage 1 and 2 active/stage 1 and 2 passive cases in year i of the data. $P_D(i)$ is the number of unstaged passive cases in year i of the data. Similarly, $A_{1M}(i), A_{2M}(i), P_{1M}(i), P_{2M}(i)$ are the number of stage 1 and 2 active/stage 1 and 2 passive cases in year i of the model. $P_D(i)$ is the number of unstaged passive cases in year i of the model. $z(i)$ is the percentage of the population screening in year i.

Metropolis Hastings MCMC was used to produce 10,000 samples with parameters drawn from the posterior parameter distribution. Few assumptions were made about the parameter distribution before fitting and so typically uninformative priors were used, the prior distributions are given in Table S7. Mean posterior estimates with 95% credible intervals obtained from fitting to data are given in Table S9.

Different variations of the model were fitted to determine the best choice of model structure. Variants were the same as those described in Rock *et al* [S13] and included ones with and without non-human animal reservoirs and different types of active screening participation in the human population (see Table S8).

Model selection was performed using the popular deviance information criterion (DIC),

$$DIC = -2LL(\bar{\theta}) + 4Var(LL(\theta)) \tag{S3.2}$$

which assigns a lower score to models with high posterior mean log-likelihood whilst penalising models with a larger number of parameters [S8]. The relative likelihood of model i was computed using,

$$\text{Relative } DIC = \exp\left((DIC_{min} - DIC_i)/2\right) \tag{S3.3}$$

and was used to compare models. As shown in Table S8, it was found that Model 5 which had both low and high-risk human groups, with some humans not participating in screening but no infected animals, was best supported by the available data. Model 8 which is the same but with animal infection, had slightly less support. Models 4 and 7 also had some support, but all other models were found to be less well supported by current data. In further analysis, the Models 4, 5, 7 and 8 were used for the analysis, weighted by their DIC score.

Table S5: Parameter notation and values used in this analysis, following Rock *et al* [S13]

Notation	Description	Value	Source
μ_H	Natural human mortality rate	5.4795 $\times 10^{-5}$ days^{-1}	[45]
B_H	Human birth rate	$= \mu_H N_H$	-
σ_H	Human incubation rate	0.0833 days^{-1}	[S15]
φ_H	Stage 1 to 2 progression rate	0.0019 days^{-1}	[S3, S4]
d_1	Passive detection rate from stage 1	-	Fitted
γ_H	Passive detection rate from stage 2	0.006 days^{-1}	Assumed
	Frequency of screening	Annual	-
	Active screen diagnostic algorithm sensitivity	0.91	Averaged from [S2]
	Active screen diagnostic algorithm specificity	1	[S2]
	Treatment compliance	1	Assumed
η_H	Pulsed active screening		
ω_H	Recovery rate	0.006 days^{-1}	[S10]
N_H	Total human population size	38674	
k_1	Proportion of low-risk, randomly participating individuals	-	Fitted
m	Relative tsetse density	$= N_V/N_H$	-
μ_V	Tsetse mortality rate	0.03 days^{-1}	[S15]
α	Tsetse bite rate	0.333 days^{-1}	[S17]
σ_V	Tsetse incubation rate	0.034 days^{-1}	[S5, S12]
p_V	Probability of tsetse infection per single infective bite	0.065	[S13]
p_H	Probability of human infection per single infective bite	-	Fitted
m_{eff}	Effective tsetse density	$= mp_H$	Fitted
ε	Reduced non-teneral susceptibility	0.05	[S13]
f_H	Proportion of blood-meal on humans	0.5	Assumed, but other values use in preliminary analysis did not impact results
r	relative risk of taken on high-risk humans compared to low-risk	-	Fitted
μ_A	Reservoir animal mortality rate	0.0014 days^{-1}	Assumed
B_A	Reservoir animal birth rate	$= \mu_A N_A$	-
σ_A	Reservoir animal incubation rate	0.0833 days^{-1}	[S15]
f_A	Proportion of blood-meals on reservoir animals	-	Fitted
N_A	Reservoir animal population size	-	Fitted
p_A	Probability of reservoir animal infection per single infective bite	-	Fitted

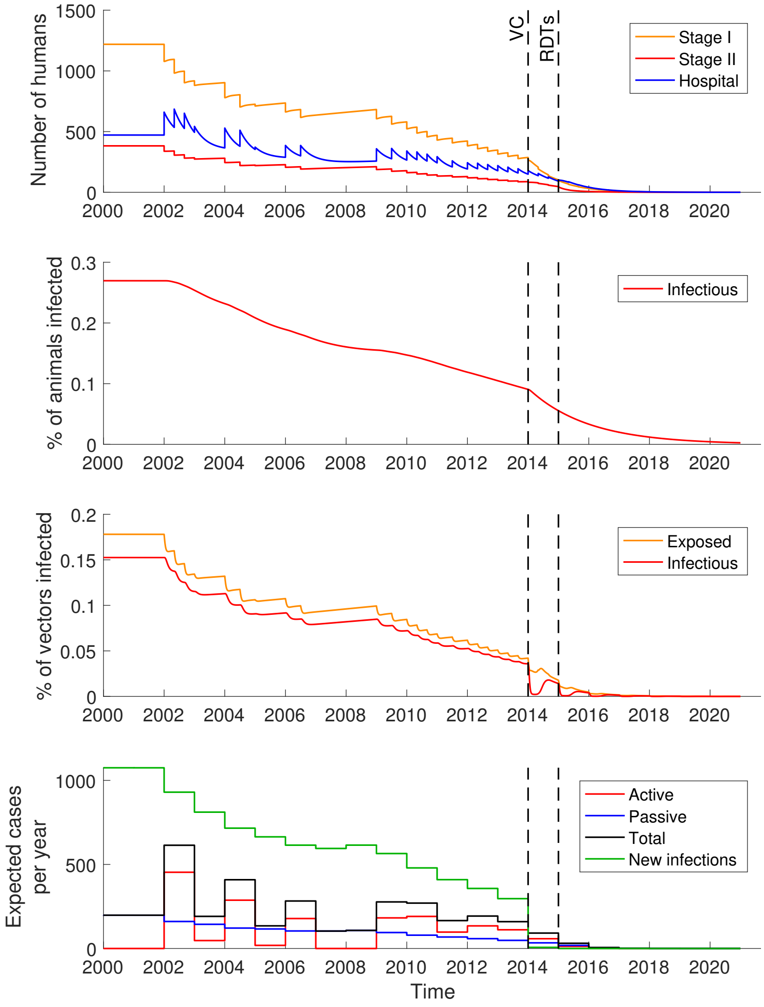

Figure S3: Example disease dynamics of the human, animal, tsetse model. The top 3 graphs show the continuous disease dynamics generated by the ODE model, with active, pulsed screening taking place from 2002 and a passive reporting level of $u = 0.22$. Additionally vector control with a 99% reduction in tsetse after 4 months is started in 2014, and improved passive detection and reporting is started in 2015. The bottom graph shows the expect number of cases and new infections which are computed after obtaining the solutions to the ODE (see (S2.3) and (S2.4)).

Table S6: Algorithm for estimating number and timing of active screening

Annual total people screened	Assumed number of screenings	Assumed screening timing
0–9,999	1	January
10,000–19,999	2	January, July
20,000+	3	January, May, September

Parameter	Prior
R_0*	U(0, ∞)
r	U(1, 100)
k_1	U(0, 1)
k_2	U(0, 1)
k_3	U(0, 1)
k_A	U(0, ∞)
f_A	U(0, 0.5)
d_1	U(0, ∞)
u	Beta(2,2)

*m_{eff} was fitted by varying R_0

Table S7: Prior distributions for target parameters

Model	Random participation		Non-participation		Animals	Relative DIC
	Low-risk	High-risk	Low-risk	High-risk		
1	X					10^{-87}
2	X	X				10^{-21}
3	X		X			10^{-23}
4	X			X		0.250
5	X	X	X	X		1
6	X				X	10^{-13}
7	X			X	X	0.129
8	X	X	X	X	X	0.683

Table S8: Different model structures under consideration and their relative DIC values in fitting using 2000–2013 data

	Model 4		Model 5		Model 7		Model 8	
	Mean	95% CI	Mean	95% CI	Mean	95% CI	Mean	95% CI
R_0	1.029949	[1.028260, 1.032260]	1.052750	[1.044050, 1.065790]	1.029149	[1.025800, 1.031890]	1.051330	[1.040890, 1.064960]
R_0^2	1.060795	[1.057319, 1.065561]	1.108283	[1.090040, 1.135908]	1.059148	[1.052266, 1.064797]	1.105295	[1.083452, 1.134140]
r	1.747933	[1.480120, 2.192525]	54.935923	[8.259660, 97.733500]	1.730117	[1.444420, 2.196685]	53.238791	[10.259400, 97.238400]
k_1	0.664222	[0.540762, 0.806268]	0.386807	[0.271427, 0.514917]	0.664711	[0.536321, 0.811379]	0.389278	[0.266366, 0.529115]
k_2	0	-	0.261779	[0.205645, 0.305139]	0	-	0.267121	[0.218550, 0.313704]
k_3	0	-	0.032780	[0.000910, 0.118150]	0	-	0.029539	[0.000800, 0.100289]
k_4	0.335778	[0.459238, 0.193732]	0.318634	[0.209325, 0.433786]	0.335289	[0.188621, 0.463679]	0.314063	[0.198777, 0.430251]
k_A	-	-	-	-	6.035863	[0.769011, 9.834170]	5.684467	[0.733206, 9.799450]
f_A	0	-	0	-	0.106916	[0.003943, 0.323657]	0.128419	[0.004618, 0.385374]
d_1	0.000467	[0.000390, 0.000552]	0.000465	[0.000389, 0.000546]	0.000465	[0.000389, 0.000553]	0.000464	[0.000387, 0.000551]
u	0.218124	[0.195748 0.244360]	0.186389	[0.166166, 0.208225]	0.220168	[0.197305, 0.246615]	0.188980	[0.167143, 0.212998]

Table S9: Parameter means and 95% credible intervals for models with moderate support from DIC

S3.1.1 Parameter uncertainty

Using MCMC allowed many parameters in the model to be estimated. These fitted parameters were the ones for which there was most uncertainty and are hard or impossible to directly measure. Other parameters, such as the tsetse bite rate and human life expectancy, have already been established directly and so these were not varied. For each of the fitted parameters, a posterior distribution was generated which captures the uncertainty in the parameter distribution. These are shown for Models 4, 5, 7 and 8 (Fig S4).

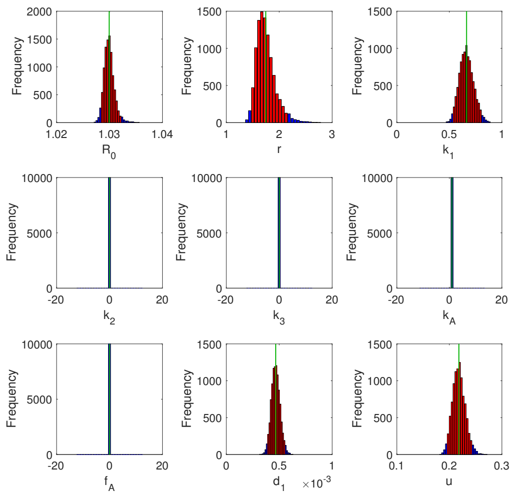

(a) Model 4

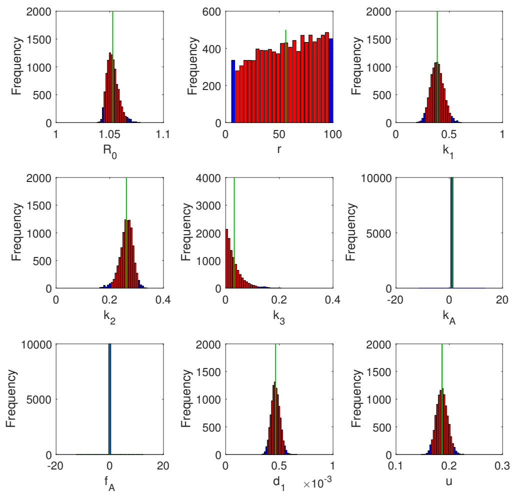

(b) Model 5

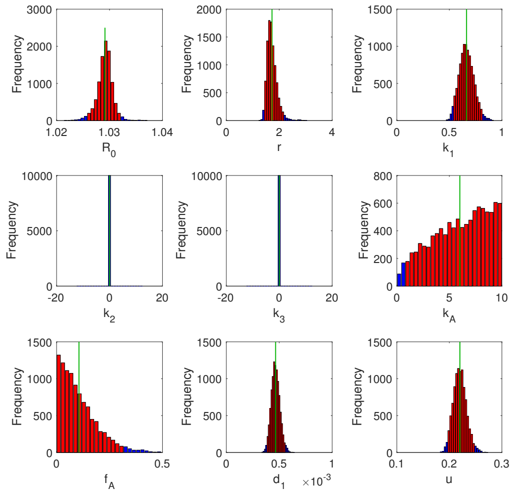

(c) Model 7

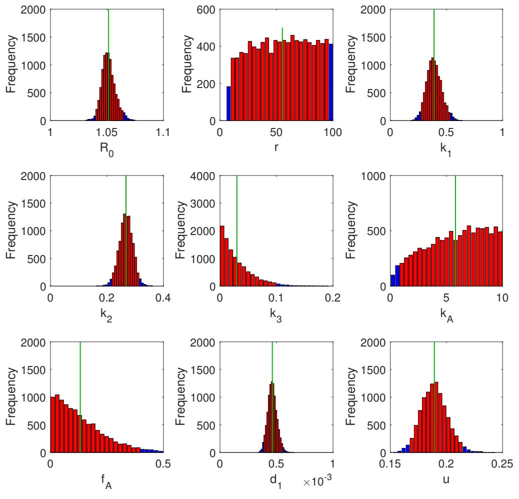

(d) Model 8

Figure S4: Posterior parameter distributions

Some parameters have quite tight posterior distributions, for example the basic reproduction number (R_0), the reporting rate from stage 1 disease (d_1) and the reporting probability (u). Under all models their mean values and credible intervals were similar.

In models with animal reservoirs (Models 7 and 8), there was large uncertainty in both the relative size of the reservoir (k_A) and frequency of tsetse bites on them (f_A) and so their posterior distribution have wide ranges. Likewise it is hard to disentangle the relative exposure of high-risk individuals from low-risk ones (r) from proportions of people in different groups if all combinations of low/high risk and participating/non-participating groups are included in the model (Models 5 and 8).

S3.2 Model validation

In order to assess the predictive ability of the model, an additional fitting round was performed using epidemiological data for the years 2000–2006 and only the number of people screened for 2007–2015. In this case, model outputs for 2007–2013 are the model's prediction for the number of cases in these years which had only medical interventions (see green box and whisker plots in Fig S5).

Analysis using DIC indicated that there is strong support for models with a mixture of high and low risk people participating/not participating in screening (Models 5 and 8) as demonstrated by the relative DIC scores (Table S10).

It is seen that the model predictions for years 2007–2015 (Fig S5) are very similar to the full model fit shown in Fig 5 (main text) and match the observed trend in the data, whilst slightly overestimating the number of active detections in some years. This demonstrates the predictive ability of the model despite some missing information such as the timing of active screenings and staging information in the early part of this period (e.g. passive detection between 2000–2004 had no staging information). As would be anticipated, credible intervals for 2014 onwards are notably larger when fewer years were used to fit the model. However, the predictions from the two fits still display similar dynamics, with any combined medical/vector control strategy leading to very low numbers of cases from 2017 onwards, compared to a slower decline under the medical-only strategies. Using a longer period of epidemiological data leads to less variation in model predictions and more confidence in short-range predictions as the data used for fitting are more recent.

Model	Random participation		Non-participation		Animals	Relative DIC
	Low-risk	High-risk	Low-risk	High-risk		
1	X					10^{-48}
2	X	X				10^{-27}
3	X		X			10^{-19}
4	X			X		0.0204
5	X	X	X	X		1
6	X				X	10^{-21}
7	X			X	X	0.0056
8	X	X	X	X	X	0.3203

Table S10: Different model structures under consideration and their relative DIC values in fitting using only 2000–2006 data

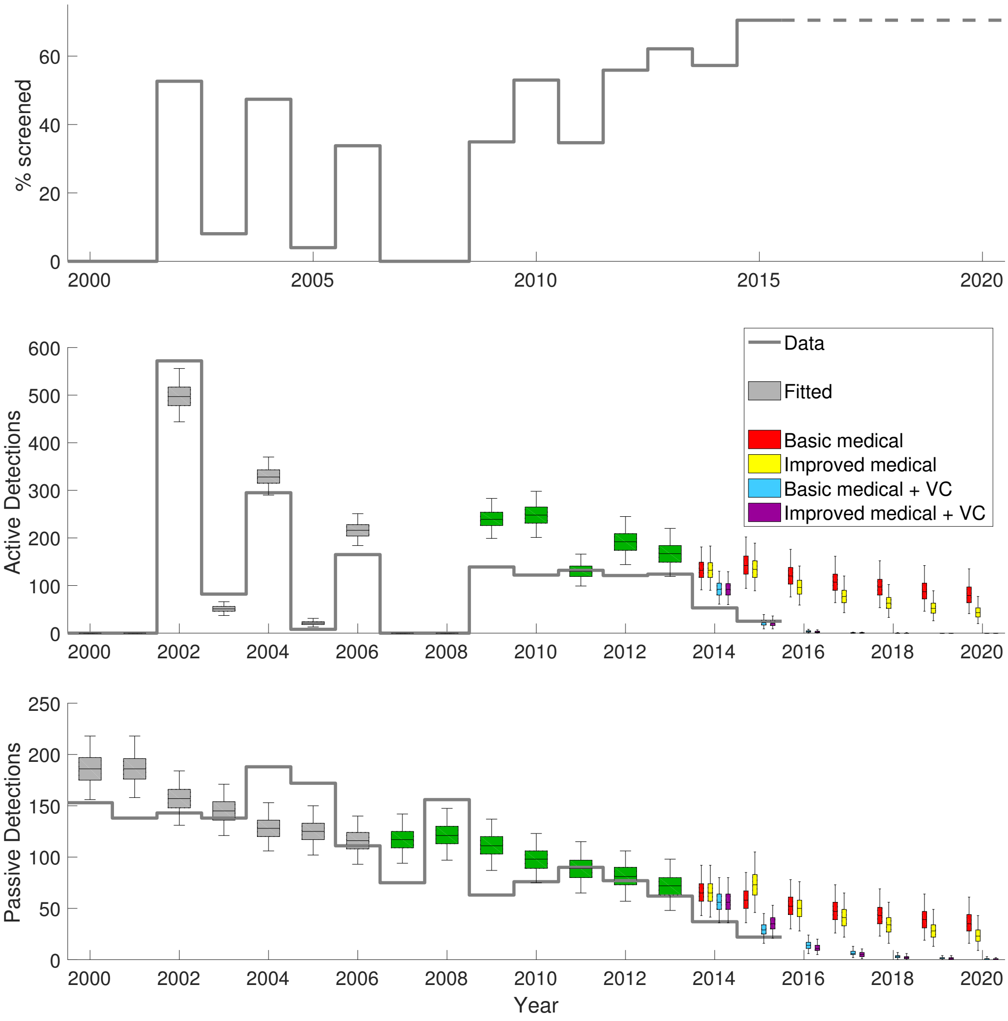

Figure S5: Grey box plots show the model fit to data from years 2000–2006. Green box plots for the years 2007–2013 are model predictions generated using the known level of active screening in each year. 2014 onwards was simulated under four strategies: (1) basic medical, using known levels of active screening and assuming passive detection continued unchanged in 2015, (2) improved medical, using known active screening and assuming passive detection and reporting rates doubled in 2015, (3) basic medical and vector control, the same as basic medical with vector control from 2014, and (4) improved medical and vector control, the same as improved medical with vector control from 2014. Strategy 4 represents the strategy that occurred in 2014–2015 and is coloured purple.

S4 Contribution to R_0

Using the Next Generation Matrix (NGM) approach [S6], a vector-host system can be analysed to determine the roles of different host types in maintaining infection. The NGM for this system (but which does not include stage 1 passive detection) is given elsewhere [S13].

Following Funk *et al* [S7], if K is the NGM, then R_0 is the spectral radius of the NGM, $\rho(K)$, and the 'host-specific' reproduction numbers for this system, U_h and U_a for humans and animals respectively, are computed using:

$$U_i = \rho((P_v + P_i)K), \qquad i \in \{h, a\}$$

with

$$P_v = \begin{pmatrix} \mathbb{0}^8 & & 0 \\ & \ddots & \vdots \\ 0 & \dots & \mathbb{1}^4 \end{pmatrix}$$

$$P_h = \begin{pmatrix} \mathbb{1}^6 & & 0 \\ & \ddots & \vdots \\ 0 & \dots & \mathbb{0}^6 \end{pmatrix}$$

$$P_a = \begin{pmatrix} \mathbb{0}^6 & & 0 \\ & \mathbb{1}^2 & \vdots \\ 0 & \dots & \mathbb{0}^4 \end{pmatrix}$$

By using parameterisation from the posterior distribution under Models 7 and 8, in 5.3% and 1.6% of parameter space respectively both $U_h < 1$ and $U_a < 1$, meaning that neither host species is classified as a maintenance host, but both are necessary for disease persistence. In the rest, $U_h > 1$ and $U_a < 1$, meaning that humans are both a maintenance host (can sustain disease without animals) and a reservoir (are necessary to have endemic disease).

References

[S1] Benjamin M Bolker, Mollie E Brooks, Connie J Clark, Shane W Geange, John R Poulsen, M Henry H Stevens, and Jada-Simone S White. Generalized linear mixed models: a practical guide for ecology and evolution. *Trends in Ecology & Evolution*, 24(3):127–135, March 2009.

[S2] F Checchi, F Chappuis, Unni Karunakara, Gerardo Priotto, and D Chandramohan. Accuracy of Five Algorithms to Diagnose Gambiense Human African Trypanosomiasis. *PLoS Neglected Tropical Diseases*, 5(7):e1233–15, July 2011.

[S3] F Checchi, J A N Filipe, M P Barrett, and D Chandramohan. The natural progression of Gambiense sleeping sickness: What is the evidence? *PLoS Neglected Tropical Diseases*, 2(12):e303, December 2008.

[S4] F Checchi, S Funk, D Chandramohan, D T Haydon, and F Chappuis. Updated estimate of the duration of the meningo-encephalitic stage in gambiense human African trypanosomiasis. *BMC Research Notes*, 8(1):292, July 2015.

[S5] S Davis, S Aksoy, and A P Galvani. A global sensitivity analysis for African sleeping sickness. *Parasitology*, 138(04):516–526, November 2010.

[S6] O Diekmann, J A P Heesterbeek, and M G Roberts. The construction of next-generation matrices for compartmental epidemic models. *Journal of The Royal Society Interface*, 7(47):873–885, April 2010.

[S7] S Funk, H Nishiura, H Heesterbeek, W J Edmunds, and F Checchi. Identifying transmission cycles at the human-animal interface: The role of animal reservoirs in maintaining Gambiense human African trypanosomiasis. *PLoS Computational Biology*, 9(1):e1002855, January 2013.

[S8] A Gelman, J B Carlin, H S Stern, D B Dunson, A Vehtari, and D B Rubin. *Bayesian Data Analysis*. CRC Press, third edition, 2013.

[S9] Paul C D Johnson, Sarah J E Barry, Heather M Ferguson, and Pie Müller. Power analysis for generalized linear mixed models in ecology and evolution. *Methods in Ecology and Evolution*, 6(2):133–142, December 2014.

[S10] A Mpanya, D Hendrickx, M Vuna, A Kanyinda, C Lumbala, V Tshilombo, P Mitashi, O Luboya, V Kande, M Boelaert, P Lefèvre, and P Lutumba. Should I Get Screened for Sleeping Sickness? A Qualitative Study in Kasai Province, Democratic Republic of Congo. *PLoS Neglected Tropical Diseases*, 6(1):e1467, January 2012.

[S11] National Climatic Data Center. US Department of Commerce. NNDC Climate Data Online. `https://www7.ncdc.noaa.gov/CDO/cdoselect.cmd?datasetabbv=GSOD`. Technical report, May 2017.

[S12] S Ravel, P Grebaut, D Cuisance, and G Cuny. Monitoring the developmental status of Trypanosoma brucei gambiense in the tsetse fly by means of PCR analysis of anal and saliva drops. *Acta Tropica*, 88(2):161–165, October 2003.

[S13] K S Rock, S J Torr, C Lumbala, and M J Keeling. Quantitative evaluation of the strategy to eliminate human African trypanosomiasis in the Democratic Republic of Congo. *Parasites & Vectors*, 8(1):1–13, October 2015.

[S14] K S Rock, S J Torr, C Lumbala, and M J Keeling. Predicting the Impact of Intervention Strategies for Sleeping Sickness in Two High-Endemicity Health Zones of the Democratic Republic of Congo. *PLOS Negl Trop ...*, pages 1–27, September 2016.

[S15] D J Rogers. A general model for the African trypanosomiases. *Parasitology*, 97:193–212, 1988.

[S16] L Thomas and C J Krebs. A review of statistical power analysis software. *Bulletin of the Ecological Society of America*, 1997.

[S17] WHO. Control and surveillance of human African trypanosomiasis. Technical Report 984, November 2013.
