# Supplementary figures and images for: Adding tsetse control to medical activities contributes to decreasing transmission of sleeping sickness in the Mandoul focus (Chad)
Source: PLoS Negl Trop Dis. 2017 Jul 27;11(7):e0005792. doi: 10.1371/journal.pntd.0005792 (PMC5549763; doi:10.1371/journal.pntd.0005792)

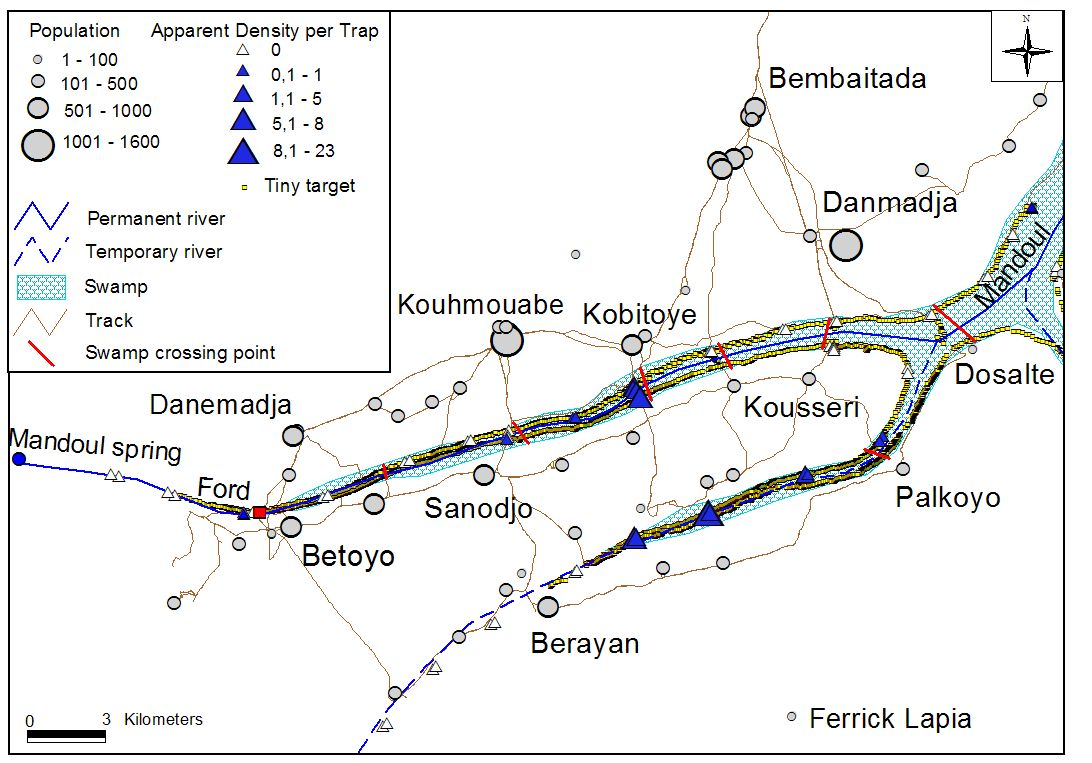

Supplement: S1 Fig — (TIF) [file pntd.0005792.s003.tif]
